# L-type calcium channels and MAP kinase contribute to thyrotropin-releasing hormone-induced depolarization in thalamic paraventricular nucleus neurons

**DOI:** 10.1152/ajpregu.00082.2016

**Published:** 2016-03-23

**Authors:** Miloslav Kolaj, Li Zhang, Leo P. Renaud

**Affiliations:** Ottawa Hospital Research Institute, Neuroscience Program and University of Ottawa, Department of Medicine, Ottawa, Ontario, Canada

**Keywords:** thyrotropin-releasing hormone, thalamic paraventricular neurons, L-type Ca^2+^ channels, TRPC-like and GIRK-like currents, mitogen-activated protein kinase

## Abstract

In rat paraventricular thalamic nucleus (PVT) neurons, activation of thyrotropin-releasing hormone (TRH) receptors enhances neuronal excitability via concurrent decrease in a G protein-coupled inwardly rectifying K (GIRK)-like conductance and opening of a cannabinoid receptor-sensitive transient receptor potential canonical (TRPC)-like conductance. Here, we investigated the calcium (Ca^2+^) contribution to the components of this TRH-induced response. TRH-induced membrane depolarization was reduced in the presence of intracellular BAPTA, also in media containing nominally zero [Ca^2+^]_o_, suggesting a critical role for both intracellular Ca^2+^ release and Ca^2+^ influx. TRH-induced inward current was unchanged by T-type Ca^2+^ channel blockade, but was decreased by blockade of high-voltage-activated Ca^2+^ channels (HVACCs). Both the pharmacologically isolated GIRK-like and the TRPC-like components of the TRH-induced response were decreased by nifedipine and increased by BayK8644, implying Ca^2+^ influx via L-type Ca^2+^ channels. Only the TRPC-like conductance was reduced by either thapsigargin or dantrolene, suggesting a role for ryanodine receptors and Ca^2+^-induced Ca^2+^ release in this component of the TRH-induced response. In pituitary and other cell lines, TRH stimulates MAPK. In PVT neurons, only the GIRK-like component of the TRH-induced current was selectively decreased in the presence of PD98059, a MAPK inhibitor. Collectively, the data imply that TRH-induced depolarization and inward current in PVT neurons involve both a dependency on extracellular Ca^2+^ influx via opening of L-type Ca^2+^ channels, a sensitivity of a TRPC-like component to intracellular Ca^2+^ release via ryanodine channels, and a modulation by MAPK of a GIRK-like conductance component.

thyrotropin-releasing hormone (TRH), initially isolated from hypothalamic tissue, is the hormone responsible for regulating the release of thyroid-stimulating hormone and prolactin from adenohypophyseal thyrotrophs and lactotrophs, respectively (reviewed in Ref. 26). The subsequent discovery of TRH-like immunoreactivity in neurons and axons in discrete extrahypothalamic regions of the brain together with evidence of TRH binding and mRNA for TRH receptors has triggered numerous investigations, which collectively suggest that TRH and its receptors contribute to a broad range of physiological functions that include arousal, thermogenesis, locomotion, analgesia, mood, and cognition (for reviews, see Refs. 26, 31, 50). In addition, potential therapeutic roles for TRH and TRH analogs are being sought in areas of neuroprotection, neuropsychiatric and mood disorders, narcolepsy, and certain forms of epilepsy (13, 50). Concurrent with these developments, investigations have sought to define the cellular mechanisms of TRH actions at its receptors in neurons, where TRH has long been considered to have a neurotransmitter role. Consistent with this notion, exogenous application of TRH has been shown to influence (usually enhance) neuronal excitability through a postsynaptic action in many central nervous system (CNS) regions. One area of current interest is the thalamic paraventricular nucleus (PVT), where our recent observations revealed that activation of TRH receptors was found to enhance neuronal excitability via concurrent decrease in a G protein-coupled inwardly rectifying K (GIRK)-like conductance and opening of a cannabinoid receptor-sensitive transient receptor potential canonic (TRPC)-like conductance (52, 53). The present study is an extension of this investigation, with a focus on the role of Ca^2+^ influx, Ca^2+^ release, and modulation by MAPK.

In endocrine tissues, TRH acts through a G protein-coupled receptor (GPCR) to promote an increase in phospholipase C (PLC) activity, an initial release of Ca^2+^ from intracellular stores and a later influx of Ca^2+^ through L-type voltage-gated Ca^2+^ channels (reviewed in Ref. 19). TRH also stimulates MAPK (33). However, in neurons, various investigations have not provided consistency in the role of Ca^2+^ entry vs. intracellular Ca^2+^ release in their response to TRH, or a relationship with MAPK. For example, in locus coeruleus, TRH-induced membrane depolarization was resistant to bath-applied nifedipine and TRH-induced inward current remained unaffected after intracellular application of BAPTA, a Ca^2+^ chelator (21). Also, in a majority of dissociated septal neurons, response to TRH was abolished in media containing nominally zero Ca^2+^, while other neurons remained responsive (43). In lateral hypothalamic orexin neurons, the TRH-induced inward current was reduced after substitution of strontium for Ca^2+^ in the bathing media (16). By contrast, the TRH-induced current in dissociated hippocampal CA1 neurons was similar to control in nominally zero Ca^2+^ media but was blocked after application of either thapsigargin in the external media or by intracellular BAPTA (10). Here, in PVT neurons in which the majority of neurons respond to exogenous TRH (52), we sought to address the role of Ca^2+^ influx vs. intracellular Ca^2+^ release, as well as the link to MAPK. We investigated the Ca^2+^ dependency of both the TRPC-like and GIRK-like components of the TRH-induced current. The results indicate a dependency on extracellular Ca^2+^ influx via L-type Ca^2+^ channels, a sensitivity of the TRPC-like component to Ca^2+^-induced Ca^2+^ release (CICR) via ryanodine channels, and a modulation by MAPK of the GIRK-like component.

## MATERIALS AND METHODS

Experimental protocols were approved by the Ottawa Hospital Research Institute Animal Care and Use Committee. We used 3–8-wk-old Wistar rats of either sex that were housed in pairs in a temperature-controlled (22–24°C) environment with a 12:12-h light-dark (LD) cycle, with food and water available ad libitum. Our earlier studies of anterior PVT neurons revealed a diurnal variation in some of their intrinsic properties, notably resting membrane potential, low threshold-activated Ca^2+^ currents (*I*_T_), hyperpolarization-activated cation currents (*I*_h_), and undefined potassium conductances (25). To minimize any diurnal variations, all animals were killed by guillotine at zeitgeber time (ZT) 2–6, during their subjective quiet-day period. After quick removal, the brain was immersed in oxygenated (95% O_2_-5% CO_2_) and cooled (<4°C) in artificial cerebrospinal fluid (ACSF) of the following composition (in mM): 127 NaCl, 3.1 KCl, 1.3 MgCl_2_, 2.4 CaCl_2_, 26 NaHCO_3_, and 10 glucose (osmolality 300–310 mosmol/kgH_2_O; pH 7.3). Brain slices 350–400 μm in thickness were cut in the coronal plane with a Vibratome 3000 Plus Sectioning System (Vibratome, St. Louis, MO), incubated in gased ACSF for >1 h at room temperature, and then transferred to a recording chamber and superfused (2–4 ml/min) with oxygenated ACSF at 32–34°C. In all experiments, the blind-patch technique was used for whole cell current-clamp and voltage-clamp recordings using either an Axopatch 1D or Axopatch 200A, respectively (Molecular Devices, Sunnyvale, CA). Data were filtered at 2 kHz, continuously monitored and stored on disk. A Digidata 1200B interface with Clampex software (pClamp 9; Molecular Devices) was used to generate current and voltage commands, as well as to store data. Recordings were obtained from PVT neurons with borosilicate thin-walled micropipettes (resistances of 3–7 MΩ) filled with (in mM) 130 K gluconate, 10 KCl, 2 MgCl_2_, 10 HEPES, 1 EGTA, 2 Mg-ATP, 0.3 Na-GTP (pH adjusted with NaOH to 7.3, osmolality ∼290 mosmol/kgH_2_O). Pipettes had resistances of 4–7 MΩ. Access resistance <15 MΩ was considered acceptable. To eliminate the contribution of Na^+^-dependent conductances, we used ACSF where NaCl was replaced with Tris-Cl (52). ACSF containing 1.3 mM barium (Ba^2+^) was utilized to eliminate the contribution of GIRK channels to TRH-induced inward currents (52). Series resistance was compensated for >65% and was regularly checked throughout the experiment by monitoring current responses to brief 5-mV hyperpolarizing steps. Basic intrinsic properties of PVT neurons in this study were consistent with those previously reported for this ZT recording time (25). Membrane voltages were corrected for liquid junction potential (normal ACSF, −13 mV; low Na^+^ ACSF, −23 mV). Voltage-clamp analyses used a standard ramp protocol (step from −63 mV to −43 mV for 0.5 s and then ramped from −43 mV to −113 mV or −123 mV in 3 s followed by step back to −63 mV) from a holding potential of −63 mV. The net TRH-induced current was obtained by subtracting the control ramp response from that obtained during the TRH-induced response. Off-line analyses were performed using Clampfit version 9 or 10 (pClamp 9 or 10; Molecular Devices). As recently reported, TRH-induced inward currents in PVT neurons rapidly desensitize (52). Therefore, the data from this study reflect responses to a single TRH application per neuron per slice. Results are expressed as means ± SE. Statistical comparisons between control and experimental values (*P* < 0.05 and better) were determined using both the paired or unpaired Student's *t*-test and ANOVA (SigmaPlot 10 and SigmaStat 3, Systat Software).

BayK8644, ω-Agatoxin IVA, thapsigargin, dantrolene, and W7 were purchased from Tocris Bioscience (R&D Systems, Minneapolis, MN). Nifedipine, BAPTA, ω-conotoxin GVIA, and PD98059 were purchased from Sigma Chemical (St. Louis, MO). Tetrodotoxin (TTX) was purchased from Alomone Labs (Jerusalem, Israel). Z941 was kindly provided by Dr. Terry Snutch from University of British Columbia, Canada. Most drugs were bath applied at the concentrations indicated.

## RESULTS

**Fig. 1.**
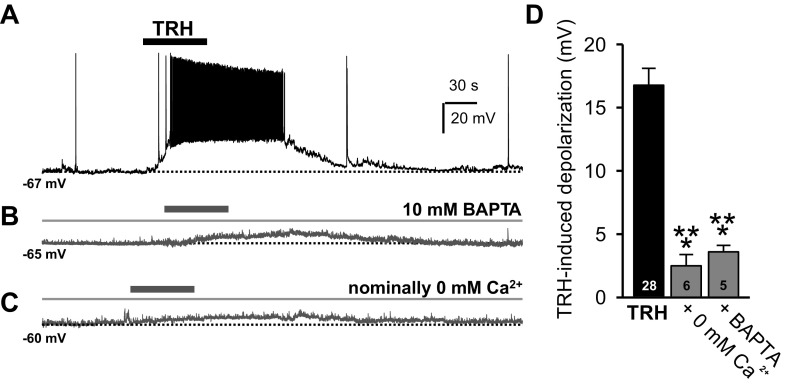
External Ca^2+^ plays a major role in thyrotropin-releasing hormone (TRH) receptor signaling. *A:* whole cell current-clamp recording illustrates the typical response of paraventricular thalamic nucleus (PVT) neurons to a 60-s bath application of 100 nM TRH (bar). *B* and *C:* whole cell current-clamp recordings from two other PVT neurons show smaller TRH responses when TRH was applied either during the internal perfusion with 10 mM BAPTA (*B*) or in artificial cerebrospinal fluid (ACSF) with nominally 0 mM Ca^2+^ (for 15 min; *C*). *D*: summary histogram illustrates TRH-induced membrane depolarization in the presence of 100 nM TRH alone (black bar; for 60 s) or when TRH was applied either during internal perfusion with 10 mM BAPTA or in ACSF with nominally 0 mM Ca^2+^ (gray bars). Data are expressed as means ± SE. ***P < 0.001.

We first evaluated the TRH response under current-clamp conditions. During intracellular dialysis with 10 mM BAPTA in the pipette media, the membrane depolarization induced by a brief bath application of TRH (100 nM for 60 s) was significantly reduced when compared with controls (Fig. 1, *A, B*, *D*; *n* = 5; one-way ANOVA, *F* = 17.024, *P* < 0.001). There was no difference in resting membrane potential between these two groups (one-way ANOVA, *F* = 2.254, *P* = 0.143). To evaluate a dependency on Ca^2+^ influx, we next used ACSF with nominally zero Ca^2+^. Here, the TRH-induced depolarization was also significantly reduced (Fig. 1, *C* and *D*; *n* = 6; one-way ANOVA, *F* = 23.705, *P* < 0.001). The data suggest major roles both for Ca^2+^ influx and intracellular Ca^2+^ in the TRH-induced response.

### 

#### TRH-induced inward current depends on Ca^2+^ entry through L-type Ca^2+^ channels.

**Fig. 2.**
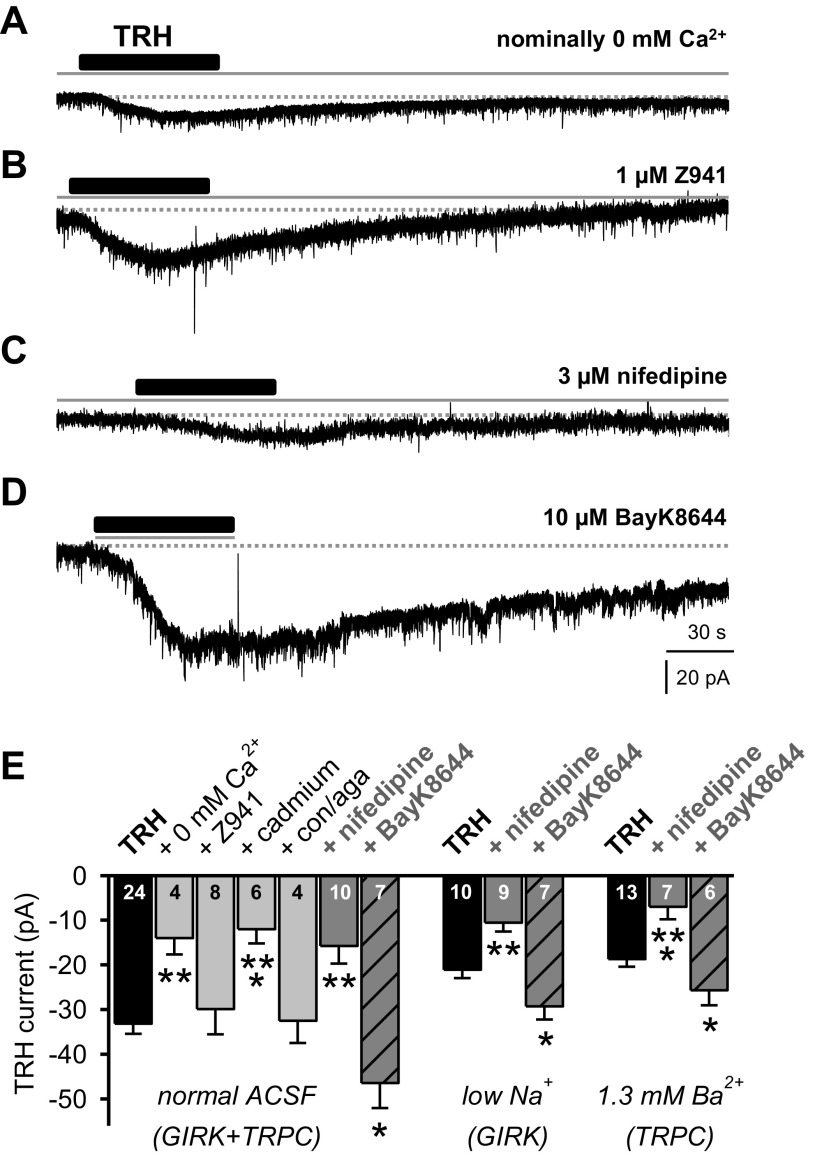
Activation of L-type Ca^2+^ channels is essential for the coupling of TRH receptor to both G protein-coupled inwardly rectifying K (GIRK)- and transient receptor potential canonical (TRPC)-like channels. Sample voltage-clamp traces done in the presence of 1 μM tetrodotoxin (TTX). *A:* typical trace showing reduced response to bath-applied TRH (100 nM for 60 s) in ACSF with nominally 0 mM Ca^2+^ (for 15 min). *B:* trace from another PVT neuron illustrates normal response to TRH in the presence of T-type Ca^2+^ channel blocker Z941 (1 μM for 10 min). *C:* trace from another PVT neuron to illustrate a reduced TRH-induced inward current in the presence of L-type Ca^2+^ channel blocker nifedipine (3 μM for 10 min). *D:* trace exhibiting both enhanced amplitude and duration of TRH-induced inward current during coadministration of L-type Ca^2+^ channel agonist BayK8644 (10 μM). *E:* summary histogram (number reflects cells tested in each condition) illustrates TRH-induced inward current either alone (100 nM; black bars) and when applied in ACSF with nominally 0 mM Ca^2+^, Z941 (1 μM), cadmium (100 μM for 10 min), ω-conotoxin GVIA (400 nM) and ω-Agatoxin IVA (con/aga; 200 nM), nifedipine (3 μM), and BayK8644 (10 μM). Note that TRH (black bars) or TRH in the presence of either nifedipine (dark gray bars) or BayK8644 (dark gray patterned bars) was applied in three different external media: control ACSF (expressing GIRK+TRPC currents), ACSF where NaCl was replaced with Tris-Cl to isolate GIRK-like channels (low Na^+^-GIRK), and ACSF with 1.3 mM Ba^2+^ to isolate TRPC-like channels (1.3 mM Ba^2+^-TRPC). Data are expressed as means ± SE. *P < 0.05; **P < 0.01; ***P < 0.001.

Recordings in voltage-clamp mode in the presence of 1 μM TTX confirmed a dependency of the TRH-induced inward current on external Ca^2+^. In ACSF containing nominally zero Ca^2+^, the response to TRH (100 nM applied for 60 s) was significantly reduced (Fig. 2, *A* and *E*; one-way ANOVA, *F* = 10.984, *P* = 0.003). Because TRH responses occurred from a holding potential around −63 mV, a voltage range at which low-voltage activated T-type Ca^2+^ channels are fully functional (see Fig. 3*A* in Ref. 25), we next evaluated the TRH-induced current in the presence of a Z941 (1 μM), a specific blocker of T-type Ca^2+^ channels (44). Whereas this treatment markedly reduced the T-type Ca^2+^ current (24), the TRH-induced current was not changed (Fig. 2, *B* and *E*; one-way ANOVA on ranks, *H* = 0.962, *P* = 0.327). However, the TRH-induced current was significantly reduced during bath application of cadmium (Cd^2+^; 100 μM), a nonselective blocker of high-voltage activated Ca^2+^ channels (HVACCs) (Fig. 2*E*; one-way ANOVA on ranks, *H* = 10.524, *P* = 0.001). While Cd^2+^ at this concentration was reported to impair T-type Ca^2+^ function in cultured thalamic neurons (34), our observations in slice preparations revealed no significant attenuation of the T-type Ca^2+^ channels in PVT neurons (see Fig. 2*C* in Ref. 55). On the basis of their pharmacological profiles, HVACCs have been further divided into N-, P/Q-, L-, and R-types (5). Because bath application of the combination of ω-Agatoxin IVA (200 nM) with ω-conotoxin GVIA (400 nM) did not affect the TRH-induced current (Fig. 2*E*; one-way ANOVA, *F* = 0.013, *P* = 0.981), a potential contribution from N-, P-, and Q-type channels would seem unlikely.

**Fig. 3.**
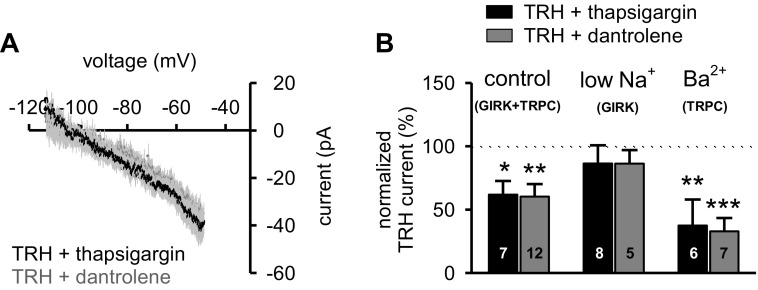
TRH-induced activation of TRPC-like channels involves Ca^2+^-induced Ca^2+^ release. *A:* the mean *I–V* relationship of the net TRH-generated current in the presence of either thapsigargin (2 μM for 15 min; *n* = 7; black circles) or dantrolene (20 μM for 15 min; *n* = 12; dark gray circles) obtained by subtracting control membrane current responses from those in TRH. Data are presented as mean (circles) ± SE (light gray lines). Note that the *I-V* reversals approximate the K^+^ equilibrium potential under these experimental conditions. *B:* summary histogram of normalized amplitude of TRH-induced currents in the presence of either thapsigargin (black bars) or dantrolene (dark gray bars) in three different external media: control ACSF (expressing GIRK+TRPC currents), ACSF where NaCl was replaced with Tris-Cl to isolate GIRK-like channels (low Na^+^-GIRK), and ACSF with 1.3 mM Ba^2+^ to isolate TRPC-like channels (1.3 mM Ba^2+^-TRPC). Note that there was no current reduction when GIRK-like channels were isolated. Data are expressed as means ± SE. *P < 0.05; **P < 0.01; ***P < 0.001; number reflects cells tested in each condition.

However, since dihydropyridine-sensitive L-type Ca^2+^ channels can be activated at low hyperpolarized voltages (28), we next assessed the response to TRH in the presence of bath-applied nifedipine (3 μM), a specific L-type Ca^2+^ channel blocker. Here, the TRH-induced inward current was reduced (Fig. 2, *C* and *E*; one-way ANOVA, *F* = 16.252, *P* < 0.001). Conversely, in the presence of BayK8644 (10 μM), an activator of L-type Ca^2+^ channels (5), the TRH-induced current was both enhanced (Fig. 2, *D* and *E*; one-way ANOVA, *F* = 6.691, *P* = 0.015) and prolonged in duration (Fig. 2*D*). These observations suggest that L-type Ca^2+^ channels contribute significantly to TRH receptor signaling in PVT neurons.

We next assessed the modulation of L-type Ca^2+^ channels with an antagonist (nifedipine) and an activator (BayK8644) under recording conditions that blocked either the contribution of TRPC-like channels (low Na^+^) or GIRK-like channels (1.3 mM Ba^2+^) to the TRH-induced response (52). As illustrated in Fig. 2*E*, both components of the TRH-induced current were reduced in the presence of nifedipine and enhanced in media containing BayK8644, suggesting that L-type Ca^2+^ channels contribute both to the closing of the GIRK-like channel component and to the opening of the TRPC-like channel component of the TRH-induced current in these neurons.

#### TRH-induced opening of TRPC-like channels relies on functional coupling between ryanodine receptors and L-type Ca^2+^ channels.

The observation that chelation of intracellular Ca^2+^ ions with BAPTA decreased TRH-induced responses suggests that Ca^2+^-dependent signaling pathways play a critical role. As we previously reported, PVT neurons display evidence of CICR (36). Therefore, we next evaluated the effect of bath-applied thapsigargin (2 μM), a noncompetitive inhibitor of sarco/endoplasmatic reticulum Ca^2+^ ATPase (SERCA). Under these experimental conditions, not only was the TRH-induced current significantly decreased (Fig. 3*B*; one-way ANOVA, *F* = 7.062, *P* = 0.013), but the net TRH current now reversed at −105 mV, close to the K^+^ equilibrium potential (Fig. 3*A*). This suggests that thapsigargin suppresses the contribution of the TRH-induced response derived from opening of TRPC-like channels. Indeed, in the presence of low Na^+^ ACSF, where the contribution from TRPC-like channels is eliminated (52), the response of PVT neurons in the presence or absence of thapsigargin was identical with control data (Fig. 3*B*; one-way ANOVA, *F* = 0.699, *P* = 0.451). However, when tested in ACSF containing 1.3 mM Ba^2+^ (to eliminate the contribution of K^+^ conductances; Ref. 52), the amplitude of the TRH-induced inward current was significantly reduced by thapsigargin (Fig. 3*B*; one-way ANOVA, *F* = 10.476, *P* = 0.005), suggesting an involvement of CICR in TRH-induced opening of TRPC-like channels.

CICR can be mediated through activation of either inositol 1, 4, 5-trisphosphate receptors (IP_3_Rs) or ryanodine receptors (RyRs). Data from our previous investigation indicated that IP_3_Rs are unlikely to be involved in the TRH-induced inward current (52, 53). However, L-type Ca^2+^ channels may be functionally coupled with RyRs (6). To address a potential L-type channel coupling with RyRs, we next bath-applied dantrolene (20 μM), a specific RyR inhibitor and obtained results that were similar to thapsigargin, i.e., the net TRH current reversed close to the K^+^ equilibrium potential (Fig. 3*A*) and the TRH-induced inward current was significantly reduced (Fig. 3*B*; one-way ANOVA, *F* = 10.389, *P* = 0.003). In addition, the TRH response was unchanged in low Na^+^ ACSF (Fig. 3*B*; one-way ANOVA, *F* = 0.864, *P* = 0.370) but significantly smaller in ACSF containing 1.3 mM Ba^2+^ (Fig. 3*B*: one-way ANOVA, *F* = 20.356, *P* < 0.001). These data suggest that CICR through functional coupling between L-type Ca^2+^ channels and RyRs contributes to the TRH-induced activation of TRPC-like channels.

#### MAPK participates in TRH-induced suppression of GIRK-like channels.

**Fig. 4.**
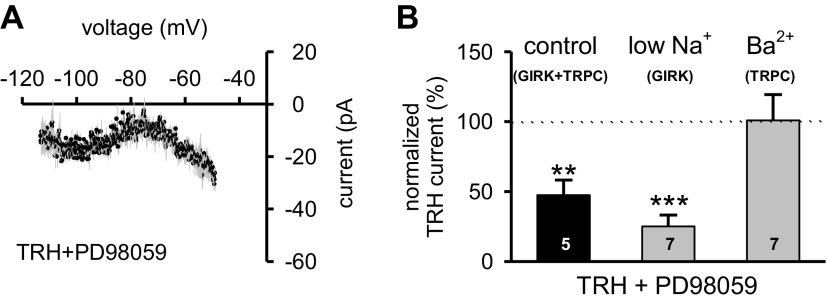
TRH-induced activation of GIRK-like channels involves MAPK. *A:* the mean *I-V* relationship of the net TRH-generated current in the presence of PD98059 (50 μM for 15 min; *n* = 5; black circles) obtained by subtracting control membrane current responses from those in TRH. Data are presented as means (black circles) ± SE (gray lines). Note that the *I-V* relationship does not display a reversal under these experimental conditions. *B:* summary histogram of normalized amplitude of TRH-induced currents in the presence of PD98059 in three different external media: control ACSF (expressing GIRK+TRPC currents; black bar), ACSF where NaCl was replaced with Tris-Cl to isolate GIRK-like channels (low Na^+^- GIRK), and ACSF with 1.3 mM Ba^2+^ to isolate TRPC-like channels (1.3 mM Ba^2+^-TRPC). Note that there was no current reduction when TRPC-like channels were isolated. Data are expressed as means ± SE. **P < 0.01; ***P < 0.001; number reflects cells tested in each condition.

Investigations in various endocrine-like cell lines reveal that activation of TRH receptors can lead to stimulation of MAPK (33, 41). To assess a possible participation of MAPK in the TRH-induced response in PVT neurons, we evaluated the impact of PD98059, a specific inhibitor of MAPK (1) on the TRH-induced current. In the presence of PD98059 (50 μM), the net TRH-induced inward current displayed no reversal potential, and the amplitude was reduced when compared with control (Fig. 4, *A* and *B*; one-way ANOVA, *F* = 8.817, *P* = 0.006), suggesting that activation of MAPK could be involved in the regulation of TRH-induced decrease of a GIRK-like conductance. In support of this, we noted that the TRH-induced current was significantly reduced when PD98059 was applied under conditions in which the activity of TRPC-like channels was inhibited (low Na^+^ data in Fig. 4*B*; one-way ANOVA, *F* = 34.764, *P* < 0.001). By contrast, when tested in ACSF containing 1.3 mM Ba^2+^, PD98059 did not alter the TRH-induced increase in TRPC-like channel conductance (Ba^2+^ data in Fig. 4*B*; one-way ANOVA, *F* = 0.002, *P* = 0.962), suggesting that MAPK only participates in the regulation of GIRK channels.

## DISCUSSION

**Fig. 5.**
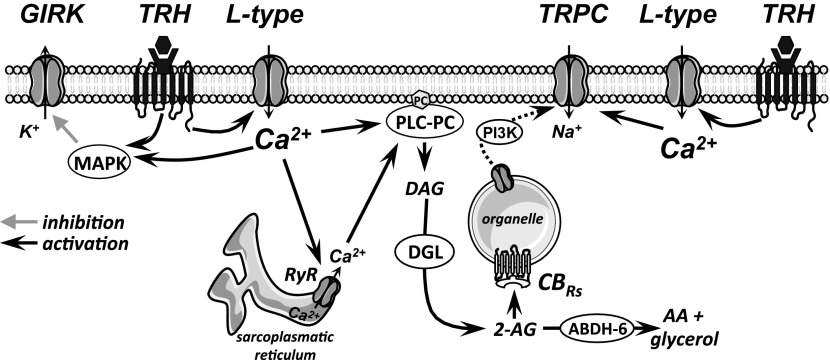
The illustration depicts an overview of the major TRH receptor signaling pathways in PVT neurons. The data are based on the current study and our recent reports on TRH receptors (52, 53).

Investigations of the TRH-induced responses in adenohypophyseal cell lines and in various expression systems have indicated that TRH-induced secretion is generally triggered by an elevation of intracellular calcium ([Ca^2+^]_i_), with a first phase of the response involving Ca^2+^ release from intracellular stores, followed by a delayed prolonged elevation of [Ca^2+^]_i_ through influx via voltage-gated Ca^2+^ channels (e.g., Ref. 19). However, in neurons, although Ca^2+^ is an important contributor to the TRH-induced responses, there is considerable diversity in terms of the role and importance of Ca^2+^ entry vs. intracellular Ca^2+^ release in different CNS regions. For example, within the diencephalon, one study using fura-2-loaded dissociated septal neurons revealed their response to TRH was either release of Ca^2+^ from internal stores, Ca^2+^ entry, or both (43). In thalamus, substitution of strontium for Ca^2+^ in the external media was noted to attenuate a TRH-induced slow afterdepolarization in lateral geniculate and perigeniculate neurons (4) and to reduce TRH-induced inward current in lateral hypothalamic orexin/hypocretin neurons (16), suggesting a role for Ca^2+^ influx. In rat PVT neurons, where there is a high density of TRH receptors (18), we recently reported that the majority of cells respond to low concentrations of exogenous TRH with an inward current that is mediated by concurrent decrease in a GIRK-like conductance and increase in a TRPC-like channel conductance, the latter linked to intracellular cannabinoid receptors (52, 53). While we have no answer as to the variability in the data on the roles of Ca^2+^ in the responses to TRH in different CNS sites, the present analysis does advance our understanding of the roles of calcium in TRH receptor signaling in PVT neurons. Collectively, the data suggest that a TRH-induced opening of L-type HVACCs is fundamental to both the TRH-induced closing of GIRK-like channels and the opening of TRPC-like channels, that the opening of TRPC-like channels is dependent on CICR, and that a MAPK participates in the closing of GIRK-like channels. Figure 5 is intended to present an overview of known TRH receptor signaling pathways in PVT neurons.

In endocrine cell lines, a role for external Ca^2+^ and for L-type Ca^2+^ channels has been well documented in the TRH receptor signaling pathway leading to hormone secretion, and an involvement of L-type HVACCs is part of TRH receptor signaling (39, 45). However, Ca^2+^ influx appears to follow CICR in endocrine cell models (reviewed in Refs. 12 and 19). By contrast, the data from the TRH-induced responses in PVT neurons suggest that influx via L-type Ca^2+^ channels is a critical early step in TRH receptor signaling, a judgment based on the following observations. First, TRH-induced depolarization is decreased in the presence of ACSF containing nominally zero Ca^2+^, attesting to the importance of extracellular Ca^2+^. Second, although TRH-induced inward current is not affected after blockade of T-type Ca^2+^ channels with Z941, sensitivity to Cd^2+^ supports an involvement of high-voltage activated Ca^2+^ channels (HVACCs). While Cd^2+^ at the concentration used here has been reported to impair T-type channel current in cultured thalamic neurons (34), we have observed no significant influence on T-type Ca^2+^ channel current in PVT neurons in our slice preparations (see Fig. 2*C* in Ref. 55). Since the TRH-induced inward current is insensitive to coapplication of ω-Agatoxin IVA/ω-conotoxin GVIA, yet modulated with nifedipine and BayK8644, the pharmacological profile seems consistent with an engagement of L-type HVACCs (5). It has been shown that nifedipine may interfere with T-type Ca^2+^ channel function (37). However, its main target appears to be the Ca_v_3.2 subtype, which has the lowest expression in PVT neurons (see Fig. 3*B* in Ref. 25). The TRH-induced response component that is mediated by the closing of GIRK-like channels is not blocked by agents acting on PC-PLC and cannabinoid receptors (53) or on CICR (Fig. 3, current study) but is almost equally as reduced as the TRPC-mediated response component in the presence of nifedipine or BayK8644 (Fig. 2*E*). Therefore, these data suggest that activation of the L-type HVACC is a foremost step in the TRH receptor signaling pathways for both closure of the GIRK-like channels and opening of the TRPC-like channels. To the best of our knowledge, an involvement of L-type HVACCs in neuronal responses to TRH has not been verified previously. Of interest, and for similarity, direct activation of L-type HVACCs by G protein-coupled receptors in CNS neurons has been reported for cholinergic receptors (54, 56), orexin receptors (23, 48), and glutamate metabotropic receptors (56, 58).

L-type Ca^2+^ channels are formed by the Ca_v_1 family, which consists of four isoforms of the α_1_ pore-forming subunit (Ca_v_1.1 to Ca_v_1.4; reviewed in Refs. 51 and 59). While L-type HVACCs are commonly activated at suprathreshold membrane potentials, a functional contribution to resting membrane potential has also been reported (29). Although there is the possibility that poor spatial clamping of dendrites may allow the voltage to be more positive and, thus, contribute to Ca^2+^ influx at remote sites, we noted minimal inward current under basal conditions at −63 mV. Recently, it has been reported that the Ca_v_1.3 gene product, in particular, can be activated at relatively hyperpolarized membrane potentials that may overlap with low-threshold T-type channels (49). Indeed, L-type Ca_v_1.3 isoforms are present in PVT (17, 42). However, our data indicate that blockade of T-type channels had no influence on the TRH-induced inward current.

Chelation of intracellular Ca^2+^ ions with BAPTA decreased the TRH-induced response, suggesting that Ca^2+^-dependent intracellular processes have a role. Consistent with this hypothesis, the data show that both thapsigargin and dantrolene decrease the TRPC-mediated component of the TRH-induced response, implying that CICR through ryanodine receptors is part of the signaling pathway leading to opening of TRPC-like channels in PVT neurons. A functional coupling between ryanodine receptors and L-type HVACCs has been established in neurons (6). Interestingly, it has also recently been suggested that there is a cross-signaling between L-type HVACCs and RyRs that control Ca^2+^ influx during neuronal activity in lateral geniculate thalamocortical relay neurons (35).

We recently reported observations suggesting that activation of TRH receptors can lead to synthesis of the endocannabinoid 2-arachidonoylglycerol (2-AG; 53). 2-AG is perhaps the most abundant endocannabinoid in the rat brain (2). Since 2-AG synthesis can depend of an increase in postsynaptic intracellular Ca^2+^ concentration (32, 38, 40), data from the current study would be in agreement with an involvement of intracellular Ca^2+^ as part of one or more pathways that lead to activation of cannabinoid receptors and consequent opening of TRPC-like channels. TRH-induced currents have a profile suggestive of TRPC4/5-like channels (52). In addition, it has been proposed that enhanced Ca^2+^ entry through either CICR or voltage-dependent Ca^2+^ channels is sufficient for TRPC5 channel activation (14). Therefore, we might speculate that Ca^2+^ ions have a dual function, one as a possible cofactor for 2-AG synthesis, another as a TRPC-like channel gating molecule.

Activation of many GPCRs leads to an increase in MAPK activity. In pituitary cells expressing endogenous TRH receptors and in a number of cell lines expressing transfected TRH receptors, exposure to TRH has been shown to stimulate MAPK (22, 33, 46). MAPK cascades can couple GPCRs to the nucleus (15), and MAPK is required for TRH stimulation of transcription of certain genes (46). Comparatively little is known with respect to a role of MAPK in TRH receptor signaling in neurons. In a study focusing on a TRH-induced increase in spontaneous inhibitory postsynaptic currents, exposure to the MAPK kinase inhibitor PD98059 reduced ongoing events to ∼60% of control but did not block TRH-induced events, suggesting that the MAPK pathway was not involved (9). Conversely, our observations in PVT neurons do suggest an involvement of MAPK in the TRH-induced current, specifically in the closure of GIRK-like channels. This was perhaps surprising given the fact that TRH responses mediated by the opening of TRPC-like channels involves activation of endogenous cannabinoid receptors (53), and MAPK is known to be part of their signaling pathways (3). On the other hand, a coupling between GIRK channels and MAPK has been suggested before (7, 27). Although we have not pursued the role of MAPK further here, it interesting to note that participation of both L-type HVACCs and MAPK are essential in prokineticin 2 receptor-mediated depolarization of neurons in the subfornical organ (11) and for estrogen receptor-induced protection of hippocampal neurons (57).

### Perspectives and Significance

Almost five decades since its initial characterization as a hormone and a neuropeptide, the physiological role of TRH in brain has remained somewhat elusive. The fact that TRH-immunoreactive axons detected in the midline thalamus likely originate from TRH-synthesizing neurons whose somata are distributed in the hypothalamus and the brain stem (20, 30, 47) suggests that endogenously released TRH may contribute in the integration of the metabolic and energy-sensing functions of the hypothalamus with the motivated behavior functions proposed for neurons in the midline thalamus. We propose that the data presented here expose a novel signaling pathway engaged by these specific TRH receptors. Whereas any systemic pharmacological manipulation that targets L-type HVACCs is likely to alter the TRH sensitivity in midline thalamic neurons, the introduction of optogenetic technology now offers the potential to selectively activate endogenous release of TRH in this thalamic location and to monitor behavioral and physiological consequences. For example, since TRH has the capability to modulate neuronal firing patterns (e.g., from burst to tonic firing), this may be a clue toward understanding some of the behavioral functions (e.g., arousal and enhanced vigilance, mood, and motivation) attributed to both TRH and to neurons in the midline thalamus (8).

## GRANTS

This research was supported by the Canadian Institutes of Health Research (CIHR) 77745. L. P. Renaud held the GlaxoSmithKline/CIHR/J. David Grimes Chair at the University of Ottawa.

## DISCLOSURES

No conflicts of interest, financial or otherwise, are declared by the authors.

## AUTHOR CONTRIBUTIONS

M.K., L.Z., and L.P.R. conception and design of research; M.K. and L.Z. analyzed data; M.K., L.Z., and L.P.R. interpreted results of experiments; M.K. and L.Z. prepared figures; M.K. and L.P.R. drafted manuscript; M.K. and L.P.R. edited and revised manuscript; M.K., L.Z., and L.P.R. approved final version of manuscript; L.Z. performed experiments.
